# Uncovering Causes of Childhood Death Using the Minimally Invasive Autopsy at the Community Level in an Urban Vulnerable Setting of Argentina: A Population-Based Study

**DOI:** 10.1093/cid/ciab838

**Published:** 2021-12-10

**Authors:** Mauricio T Caballero, Sebastian Diaz Grigaites, Paola X De la Iglesia Niveyro, Sebastian Esperante, Alejandra M Bianchi, Alejandra Nuño, Sandra Valle, Gabriela Afarian, Adrian J P Ferretti, Sofia Jares Baglivo, Julian De Luca, Cristian M Zea, Paula Caporal, Maria Jose Labanca, Adriana Diamanti, Damian Alvarez-Paggi, Quique Bassat, Fernando P Polack, Community Mortality Network, Ana M Carballo, Gabriela Hernandez, Paola Figueroa, Patricia E Ares, Cesar A Rodriquez Paquete

**Affiliations:** 1 Fundacion INFANT, Buenos Aires, Argentina; 2 Consejo Nacional de Investigaciones Científicas y Técnicas, Buenos Aires, Argentina; 3 Morgue Judicial del Instituto de Ciencias Forenses Conurbano Sur, Ministerio Público de la Provincia de Buenos Aires, Lomas de Zamora, Argentina; 4 Hospital Italiano de Buenos Aires, Servicio de Anatomía Patológica, Buenos Aires, Argentina; 5 Hospital Juan P Garrahan, Buenos Aires, Argentina; 6 Hospital De Niños Sup. Sor Maria Ludovica, La Plata, Argentina; 7 ISGlobal, Hospital Clínic–Universitat de Barcelona, Barcelona, Spain; 8 Centro de Investigação em Saúde de Manhiça, CP Maputo, Mozambique; 9 Institución Catalana de Investigación y Estudios Avanzados (ICREA), Pg. Lluís Companys, Barcelona, Spain; 10 Pediatric Infectious Diseases Unit, Pediatrics Department, Hospital Sant Joan de Déu, University of Barcelona, Barcelona, Spain; 11 Consorcio de Investigación Biomédica en Red de Epidemiología y Salud Pública, Madrid, Spain

**Keywords:** community mortality, minimally invasive tissue sampling, population-based surveillance study

## Abstract

**Background:**

Precise determination of the causal chain that leads to community deaths in children in low- and middle-income countries is critical to estimating all causes of mortality accurately and to planning preemptive strategies for targeted allocation of resources to reduce this scourge.

**Methods:**

An active surveillance population-based study that combined minimally invasive tissue sampling (MITS) and verbal autopsies (VA) among children under 5 was conducted in Buenos Aires, Argentina, from September 2018 to December 2020 to define the burden of all causes of community deaths.

**Results:**

Among 90 cases enrolled (86% of parental acceptance), 81 had complete MITS, 15.6% were neonates, 65.6% were post-neonatal infants, and 18.9% were children aged 1–5 years. Lung infections were the most common cause of death (CoD) in all age groups (57.8%). Among all cases of lung infections, acute bronchiolitis was the most common CoD in infants aged <12 months (23 of 36, 63.9%), and bacterial pneumonia was the most common cause in children aged >12 months (8 of 11, 72.7%). The most common comorbid condition in all age groups was undernutrition in 18 of 90 (20%). It was possible to find an immediate CoD in 78 of 81 subjects where MITS could be done. With this combined approach, we were able to determine that sudden infant death syndrome was overestimated in state reports.

**Conclusions:**

CoD determination by a combination of MITS and VA provides an accurate estimation of the chain of events that leads to death, emphasizing possible interventions to prevent mortality in children.

Although global child mortality has decreased by nearly two-thirds in the last decades, there are regions in the developing world where mortality rates are still unacceptably high [1, 2]. Cause of death (CoD) estimations are key to planning and prioritizing efficient health interventions that can be rapidly scaled up in low-resource settings [3, 4]. However, the scarcity of primary source data and the poor quality of this information make CoD estimations a problem in low- and middle-income countries (LMICs) where most of the deaths occur [3-6]. Indeed, home deaths are frequent and “hard to reach” in LMICs, where these fatalities are commonly attributed through a verbal autopsy (VA) method, which can be very deficient and biased in identifying the causal chain that leads to fatal outcomes [7-10].

Community-based mortality ascertainment in LMICs is challenging because many deaths go undetected by state surveys or by facility-based audit systems [7, 9, 10]. In addition, postmortem diagnosis based on complete diagnosis autopsies are rare; families usually reject postmortem methods based on their cultural beliefs; and some burials occur quickly after death, hindering sampling procedures [5, 10-13]. Minimally invasive tissue sampling (MITS) is a well-accepted, less disruptive, and faster method that was developed to enhance the precise ascertainment of CoD and to close the gaps in postmortem procedures [8, 11, 14]. This technique, used in combination with molecular biology and standard CoD determination by a panel of experts, leads to a more thorough investigation of the etiological diagnosis, opening up a new range of possibilities for active disease surveillance [8, 15, 16].

Our group built a population-based child community mortality surveillance platform in a very deprived and poorly resourced region of Buenos Aires since 2016 [7]. We found that some babies who died at home in circumstances of high structural poverty were infected in the nasopharynx by respiratory pathogens [7]. Between 2018 and 2020, we expanded our program to a multidisciplinary approach based on MITS, VAs, molecular techniques, and standardized CoD attribution to determine the cause of home deaths [17].

## METHODS

### Study Design and Approach

We designed an active surveillance population-based prospective study in collaboration with a judicial morgue that received children, on a daily basis, who died at home, “on arrival” to a health facility, or in a regional hospital in the context of medical malpractice [17]. The study was conducted from September 2018 to December 2020 in a catchment population of 40 027 yearly live births in 6 districts of the Province of Buenos Aires, Argentina. Inclusion criteria included all under 5 children who died at home (outside any health facility), those certified as dead on arrival by a physician at a hospital, and those deaths classified as unusual, suspicious, or of unknown cause whereby a local prosecutor requested a full necropsy. Deaths in children with a criminal or external underlying cause were excluded for legal reasons.

Study coordinators screened all child deaths for study eligibility and enrollment. Trained social workers, psychologists, pediatricians, or study researchers contacted families for informed consent, and grief counseling was provided in a separate room used for this purpose [17]. All families were also advised of the state’s bureaucratic procedures related to the child’s death. For subjects included in the study, MITS was conducted within 24–48 hours after death; all bodies were refrigerated until sampling was performed [8, 14]. Lung, liver, heart, brain, blood, and rectal samples and nasopharyngeal swabs were obtained and frozen at –80°C [8]. Additional tissue samples were fixed for 6–48 hours in formaldehyde 10% buffer. All samples were screened using quantitative polymerase chain reaction (qPCR) for 20 prevalent pathogens, and tissues were stained with hematoxylin-eosin [7, 18]. Specific immunohistochemistry was conducted for respiratory syncytial virus (RSV), cytomegalovirus (CMV), and influenza A. Between 30 and 90 days after death, a team composed of a social worker and a pediatrician interviewed the parents or direct relatives to obtain clinical and epidemiological data (VA) using the standard 2016 VA tool [7]. After all procedures were performed, a CoD was attributed by an expert panel following Determination of Cause of Death (DeCoDe) standards [8, 15, 19]. All data were stored in REDCap [20, 21]. The state’s institutional review board approved the study, and the Justice Department endorsed the study through an interinstitutional collaboration agreement [7].

### Tissue Sample Collection

The MITS technique was performed as previously described [8, 14, 17]. At least 2 previously trained individuals were involved in the procedures [8, 14]. Percutaneous tissue samples were taken using an automatic biopsy gun for Tru-Cut needles (DANA 2.2 MG, HISTO) and 12- to 14-gauge disposable core biopsy needles Tru-cut type (BIOCORE II MG, HISTO). All procedures were carried out with the use of adequate personal protection equipment and following biosafety guidelines (biosafety level 2) [8, 14]. A previously designed specimen collection form was completed during all MITS.

### Nucleic Acid Purification

All tissue samples targeted for microbiological analyses were stored at −80°C until DNA/RNA purification. Tissue cores obtained using MITS were lysated at 55°C using a dry block heater (Thermo Scientific™) in 200 µL of tissue lysis buffer (Qiagen), 20 µL of 10 mg/mL proteinase K (Qiagen), and 5 µL β-mercaptoethanol (Sigma-Aldrich). Tissue samples were homogenized by vortexing during lysis. Nucleic acid extractions (DNA + RNA) were performed using a Pathogen’s DNA/RNA purification kit (Applied Biosystems™) in a semiautomated system (MagMAX™ Express-96 Deep Well Magnetic Particle Processor). All samples were analyzed for nucleic acid concentration (nanograms per microliter) and purity (260/280 absorbance ratio) using a full spectrum (220–750 nm) spectrophotometer (Thermo Scientific).

### Determination of the CoD

The study team generated report forms for each procedure (necropsy, histopathology, microbiology, VA, and clinical records) for all enrolled subjects who were available for CoD determination [8, 15, 17, 19]. Two panels of experts trained in death certification and the DeCoDe process reviewed the collection forms independently to establish the most probable causes and chain of events leading to death [15, 17]. For the purpose of the study, a death certificate was created for each subject using a mortality classification system derived from the *International Statistical Classification of Disease and Related Health Problems, Eleventh Revision*, from the World Health Organization (*International Classification of Diseases, Eleventh Revision, Clinical Modification*); for perinatal cases, a specific version was used (ICD-Perinatal [PM]) [22].

### Statistical Analyses

Population live birth information was derived from the state regional surveillance system in the catchment area to estimate community mortality rates [7, 23]. Frequency distribution, mean, median, interquartile range (IQR), standard deviation, and 95% confidence interval (CI) were used for variable description as appropriate. Cohen’s kappa coefficient was used to measure DeCoDe interrater reliability between expert panels; the agreement scale was categorized as slight (<0.2), fair (0.21–0.40), moderate (0.41–0.60), substantial (0.61–0.80), and almost perfect (>0.80) [24]. A *P* value < .05 was considered statistically significant. Statistical analyses were performed using Stata version 15 (StataCorp).

## RESULTS

### Burden of Community Child Mortality in a Low-Resource Region of Argentina

**Figure 1.**
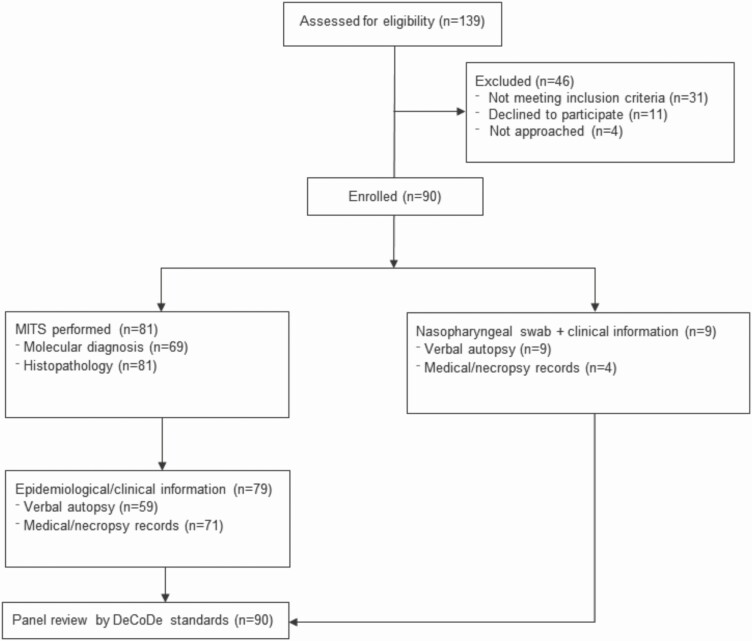
Flow diagram of study process. Abbreviations: DeCoDe, Determination of Cause of Death; MITS, minimally invasive tissue sampling.

**Figure 2.**
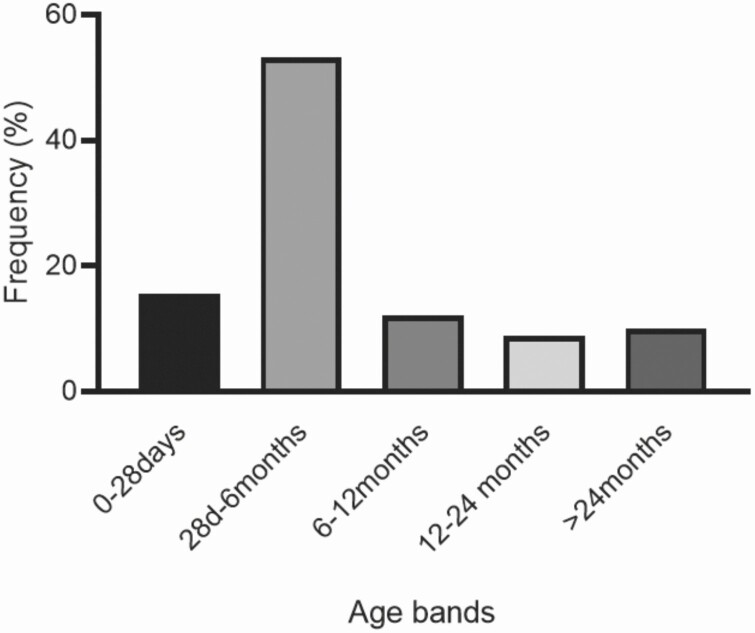
Frequency of age distribution for all deaths stratified by age group.

From September 2018 to December 2020, our program screened a susceptible population of nearly 250 000 children aged <5 years living in 6 counties in the southern outskirts of Buenos Aires, Argentina. We included 90 deaths from children aged <5 years who died at home, on arrival at any health facility in the region, or died at a hospital and a prosecutor ordered a full necropsy for legal reasons (Figure 1). Eighty-six percent of eligible subjects were included after parental consent. MITS was performed for 81 bodies (90%); histopathological samples were reviewed and informed by 2 independent pathologists (kappa = 0.56, *P* < .001). The majority of fatalities occurred at home or on the way to a health facility (86.7%), and few of them (8.9%) occurred up to 12 hours after hospital admission (most were in the emergency department). Only 4 infant deaths (4.4%) took place while hospitalized, where full necropsies were completed per instruction of a state attorney. Deaths most frequently occurred in the post-neonatal period (84.2%; 95% CI, 70–88), with a median age of 2.98 months and a mean of 7.7 months (IQR, 1.5–7.6; Figure 2). As previously reported, we found that most families of deceased children lived in conditions of structural poverty with household crowding (92%), in homes located in slums and settlements (80%), where the proportion unemployed parents was high (76%), where parental education was incomplete (71%), and where recent stressful situations had occurred (39%) [7, 18].

### CoD Attribution by DeCoDe Expert Panels

Immediate and underlying causes of death were determined by 2 independent DeCoDe expert panels that evaluated all available information and, using a diagnosis standard, sought consensus to attribute the most likely chain of events leading to death in cases where MITS was conducted [15, 17, 19, 25]. In cases for which MITS was not conducted, the CoD determination was performed by assessing qPCR of nasopharyngeal swabs, personal disease records, perinatological records, and the death event’s report. The attribution of CoD by the 2 independent expert panels showed a substantial diagnostic concordance (kappa = 0.69, standard error = 0.15, *P* < .001).

Overall, at least 1 immediate or underlying CoD was identified in 83 of 90 (92.2%) cases; in instances where MITS was not performed, the proportion of CoD assignment was 66%. The most common underlying causes of deaths in all age groups were lung diseases (66.7%). In fact, we found that nearly 70% of all deaths had any lung illness by histopathology (Table 1). Lung infections were the most common respiratory disease, and we found 82% of respiratory organisms by qPCR in lungs. The pathogen most frequently found in lung tissue samples was *Streptococcus pneumoniae*, being present in the causal chain that led to death of 18.9% of cases. In infants, a pattern of mixed bronchiolitis/pneumonia histopathology was found in more than 50% of cases. RSV was the major cause of mortality in 2019 (Table 2). However, we did not find RSV in 2018 and 2020. Possible explanations for this phenomenon are that enrollment began in spring 2018 after the RSV season and the 2020 lockdown due to coronavirus disease 2019 affected RSV circulation worldwide [26, 27]. In children aged 12–59 months, lung infections accounted for 64.7% of CoD attributions, where bacterial pneumonia was the most frequent and severe pathogen present (72.7%). Other pathogens that were found included *Staphylococcus aureus* (5.6%), *Bordetella pertussis* (3.3%), influenza A (2.2%), *Klebsiella pneumoniae* (4.4%), *Pseudomona aeruginosa* (2.2%), CMV (2.2%), adenovirus (2.2%), and rhinovirus (2.2%). We also found many cases with *Moraxella catarrhalis* in respiratory samples; however, its role in pathogenesis was unclear for both expert panels [28]. Surprisingly, we found pneumonitis due to milk or food aspiration in 17.8% of immediate causes of home deaths (Table 1). Most cases were accompanied by mild bronchiolitis or pneumonia; infants aged <6 months were most affected (median age, 3.5 months; IQR, 2.9–4.1), and 93.7% of the cases were male infants (*P* = .004). Lung coinfection was common in our study (46%), mostly in children with comorbidities or chronic lung diseases.

**Table 1.** Frequency of Immediate and Underlying Causes of Community Deaths by the Expert Panel Determination of Cause of Death

Immediate and Underlying Cause of Death	Total, n = 90 n/N (%)	Neonates, n = 14 n/N (%)	Infants, n = 59 n/N (%)	Children, n = 17 n/N (%)
Lung infections	54/90 (60.0)	5/14 (35.7)	38/59 (64.4)	11/17 (64.7)
Bacterial pneumonia	31/54 (57.4)	2/5 (40.0)	21/38 (52.8)	8/11 (72.7)
Acute bronchiolitis	27/54 (50.0)	2/5 (40.0)	23/38 (63.9)	2/11 (18.2)
Acute bronchitis	3/54 (5.6)	0/5 (0.0)	3/38 (8.8)	0/11 (0.0)
Abscess of lung	1/54 (1.8)	0/5 (0.0)	1/38 (2.8)	0/11 (0.0)
Pyothorax	1/54 (1.8)	0/5 (0.0)	0/38 (0.0)	1/11 (9.1)
Tuberculosis	2/54 (3.7)	0/5 (0.0)	2/38 (5.3)	0/11 (0.0)
Influenza	2/54 (3.7)	1/5 (20.0)	0/38 (0.0)	1/11 (9.1)
Other respiratory diseases	23/90 (25.5)	2/14 (14.3)	16/59 (27.1)	5/17 (29.4)
Pneumonitis due to inhalation of food or vomit	16/23 (69.6)	2/2 (100)	13/16 (81.2)	1/5 (20.0)
Chronic lung diseases	7/23 (30.4)	0/2 (0.0)	3/16 (18.8)	4/5 (80.0)
Sepsis	4/90 (4.4)	0/14 (0.0)	2/59 (3.4)	1/17 (5.9)
Meningitis or encephalitis	6/90 (6.6)	0/14 (0.0)	6/59 (10.2)	0/17 (0.0)
Congenital birth defects	5/90 (5.5)	2/14 (14.3)	2/59 (3.4)	1/17 (5.9)
Congenital infection	3/90 (3.3)	1/14 (7.1)	2/59 (3.4)	0/17 (0.0)
Human immunodeficiency virus	1/90 (1.1)	0/14 (0.0)	1/59 (2.9)	0/17 (0.0)
Asphyxia	5/90 (5.5)	2/14 (14.3)	0/59 (0.0)	4/17 (23.5)
Birth complications	3/90 (1.1)	0/14 (0.0)	1/59 (2.9)	2/17 (11.8)
Heart diseases	7/90 (7.8)	3/14 (21.4)	4/59 (6.8)	0/17 (0.0)
Preterm complications	1/90 (1.1)	0/14 (0.0)	1/59 (2.9)	0/17 (0.0)
Perinatal asphyxia or hypoxia	2/90 (2.2)	0/14 (0.0)	0/59 (0.0)	2/17 (11.8)
Diseases of the nervous system	3/90 (3.3)	1/14 (7.1)	0/59 (0.0)	2/17 (11.8)
External causes	5/90 (5.6)	0/14 (0.0)	3/59 (5.1)	2/17 (11.8)
Sudden infant death syndrome	7/90 (7.8)	1/14 (7.1)	6/59 (10.2)	0/17 (0.0)
Other significant conditions				
Lung infections	3/90 (2.2)	0/14 (0.0)	2/59 (3.4)	1/17 (5.9)
Other respiratory disease	3/90 (2.2)	1/14 (7.1)	3/59 (5.1)	1/17 (5.9)
Congenital syphilis	6/90 (6.7)	2/14 (14.3)	3/59 (5.1)	1/17 (5.9)
Undernutrition	18/90 (20.0)	2/14 (14.3)	12/59 (20.3)	4/17 (23.5)
Underweight	14/18 (77.8)	2/2 (100)	9/12 (75.0)	3/4 (75.0)
Wasting	4/18 (22.2)	0/2 (0.0)	3/12 (25.0)	1/4 (25.0)
Congenital birth defects	9/90 (10.0)	1/14 (7.1)	5/59 (8.5)	3/17 (17.6)
Liver disease	7/90 (7.8)	1/14 (7.1)	4/59 (6.8)	2/17 (11.8)
Heart diseases	4/90 (4.4)	0/14 (0.0)	3/59 (5.1)	1/17 (5.9)
Preterm	6/90 (6.7)	0/14 (0.0)	4/59 (6.8)	2/17 (11.8)
Palliative care	4/90 (4.4)	0/14 (0.0)	2/59 (3.4)	2/17 (11.8)

**Table 2.** Frequency of Pathogens Identified in All Tissue Samples and in Causal Chain Leading to Death Based on Quantitative Polymerase Chain Reaction

Pathogen	Total (%)	Causal Chain (%)	Nasopharyngeal Swab (%)	Lungs (%)	Blood (%)	Brain (%)	Liver (%)
*Streptococcus pneumoniae*	29/90 (32.2)	17/90 (18.9)	28/90 (31.1)	17/90 (18.9)	3/90 (3.3)	0/90 (0.0)	1/90 (1.1)
Rhinovirus	24/90 (26.7)	2/90 (2.2)	24/90 (26.7)	1/90 (1.1)	1/90 (1.1)	0/90 (0.0)	0/90 (0.0)
*Moraxella catarrhalis*	21/90 (23.3)	4/90 (4.4)	20/90 (22.2)	16/90 (17.8)	0/90 (0.0)	0/90 (0.0)	0/90 (0.0)
Respiratory syncytial virus	13/90 (14.4)	11/90 (12.2)	6/90 (6.7)	13/90 (14.4)	2/90 (2.2)	0/90 (0.0)	2/90 (2.2)
*Staphylococcus aureus*	13/90 (14.4)	5/90 (5.6)	11/90 (12.2)	8/90 (8.9)	0/90 (0.0)	0/90 (0.0)	0/90 (0.0)
Cytomegalovirus	13/90 (14.4)	3/90 (3.3)	13/90 (14.4)	3/90 (3.3)	2/90 (2.2)	0/90 (0.0)	0/90 (0.0)
*Klebsiella pneumoniae*	7/90 (7.8)	4/90 (4.4)	0/90 (0.0)	4/90 (4.4)	1/90 (2.2)	1/90 (1.1)	0/90 (0.0)
*Pseudomona aeruginosa*	4/90 (44.4)	2/90 (2.2)	0/90 (0.0)	2/90 (2.2)	0/90 (0.0)	0/90 (0.0)	0/90 (0.0)
*Bordetella pertussis*	3/90 (3.3)	3/90 (3.3)	3/90 (3.3)	3/90 (3.3)	0/90 (0.0)	0/90 (0.0)	0/90 (0.0)
Haemphilus influenzae B	3/90 (3.3)	1/90 (2.2)	3/90 (3.3)	1/90 (2.2)	0/90 (0.0)	0/90 (0.0)	0/90 (0.0)
Influenza A	2/90 (2.2)	2/90 (2.2)	2/90 (2.2)	2/90 (2.2)	1/90 (1.1)	0/90 (0.0)	0/90 (0.0)
Adenovirus	2/90 (2.2)	2/90 (2.2)	0/90 (0.0)	2/90 (2.2)	0/90 (0.0)	0/90 (0.0)	0/90 (0.0)
Human immunodeficiency virus	1/90 (1.1)	1/90 (1.1)	0/90 (0.0)	0/90 (0.0)	1/90 (1.1)	0/90 (0.0)	0/90 (0.0)
Parainfluenza virus 3	1/90 (2.2)	1/90 (2.2)	1/90 (2.2)	1/90 (2.2)	0/90 (0.0)	0/90 (0.0)	0/90 (0.0)
*Mycobacterium tuberculosis*	2/90 (2.2)	0/90 (0.0)	2/90 (2.2)	0/90 (0.0)	0/90 (0.0)	0/90 (0.0)	0/90 (0.0)

Severe bacterial infection was found in 11 children (12.2%). However, in those with central nervous system infection, focal histopathological disease was found, and in only 1 case was there a microbiological finding. Bacterial sepsis was always associated with the finding of bacterial pneumonia. Despite the suspicion of sepsis in multiple cases, we were able to detect a pathogen in only 4 patients. Several respiratory viruses were found in blood samples (RSV, 2 cases; influenza, 1 case; CMV, 2 cases; and rhinovirus, 1 case). Numerous congenital diseases (24%) were found in the causal chain that led to death, including birth defects (5.5%) and complications (1.1%), infections (4.4%), heart diseases (7.8%), and preterm complications (1.1%).

During the study period, we found that almost 30% of infant deaths were attributed to sudden infant death syndrome (SIDS) as the underlying CoD. However, after the DeCoDe process, experts were able to recognize other fatal illnesses and assigned SIDS as an immediate CoD in just 7 cases. Nevertheless, in 9 cases, the CoD attribution was difficult because of the lack of tissue samples, and we considered that perhaps SIDS was overestimated.

Other significant conditions were found in 48 of 90 (53%) subjects enrolled. The most frequent comorbidity detected was undernutrition (20%), and 8 of 18 (44%) were severe. Sixty-six percent of the malnourished children died due to lower respiratory tract infections (LRTI), and 22% were chronically affected. Perinatal diseases were also important conditions related to deaths. We found 26% of diseases: birth defects (10%), heart diseases (4%), congenital syphilis (6%), and history of prematurity (6%) with admission in neonatal intensive care units.

### Immediate and Underlying CoD Attribution Using VA Compared With MITS and Full Necropsy

Among 81 subjects with complete MITS, 59 had additional individual information from a completed VA instrument. In LMICs, where many deaths occur at the community level, a significant proportion of CoD attribution is made using VA as a unique instrument to inform CoD. Therefore, we wanted to determine how accurate VA was in discriminating between immediate and underlying CoD in our population. VA performance information can be relevant for community mortality estimates, understanding parental awareness regarding diseases, and identifying delays in reaching health care.

VA had fair agreement with MITS when identifying LRTI in all subjects (kappa = 0.39, *P* < .001), while sensitivity and specificity analysis were 56% and 90%, respectively (Table 3). When we analyzed by pathogen, we found that sensitivity and specificity for pneumonia due to *S. pneumoniae* were 54% and 63%, respectively, and were very similar for RSV and LRTI with 58% sensitivity and 64% specificity. We then evaluated for other CoD and found that for all infections, VA also had a fair agreement (kappa = 0.22, *P* = .033) and 47% of sensitivity and 77% of specificity. However, there was no agreement in sepsis or meningitis diagnosis between the 2 methods (kappa = –0.11, *P* = .82). Agreement for heart disease between both approaches was moderate (kappa = 0.44, *P* < .001); the sensitivity was 60% and the specificity was 93%. Together, the VA instrument had fair agreement for CoD attribution compared with MITS plus necropsy and had moderate agreement for heart disease identification (Table 3).

**Table 3.** Performance of Verbal Autopsy Compared With Minimally Invasive Tissue Sampling Plus Necropsy at the Individual Level to Assign Cause of Death

	Verbal Autopsy Agreement						
Minimally Invasive Tissue Sampling + Necropsy	Agreement n/N (%)	Kappa	*P* Value	Sensitivity	Specificity	Positive Predictive Value	Negative Predictive Value
Lower respiratory tract infections by determination of cause of death	20/36 (55.6)	0.39	<.001	0.56	0.90	0.91	0.53
All infections	16/34 (47.1)	0.22	.033	0.47	0.77	0.76	0.49
Sepsis or meningitis	0/5 (0.0)	-0.11	.823	0.00	0.85	0.00	0.90
Congenital heart disease	3/5 (60.0)	0.44	.001	0.60	0.93	0.43	0.96

## Discussion

In this study, we implemented an active surveillance population-based study to determine all causes of community deaths in a low-income region of Buenos Aires, Argentina, using a combined approach of MITS, VA, molecular biology, and experts’ case review. Using this approach, we were able to assign an immediate or underlying CoD in 92% of cases. Overall, 86% of parents consented to participate in the study; however, the proportion of VA acceptance was lower (75%). Despite the high rate of acceptance, we considered that the legal circumstances regarding home deaths in Argentina may have played a role in parents’ willingness to participate. Additionally, we compared the performance of the VA instrument with MITS plus necropsy as the gold standard. Among all causes analyzed, VA diagnosis concordance with MITS was often fair to moderate across most common group of illnesses at the individual level.

As we previously described, home fatalities of children aged <5 years living in vulnerable settings in Buenos Aires’ occur mainly in post-neonatal infants. Consequently, the main group of diseases associated with mortality are those prevalent in this age group, that is, LRTI and infectious diseases [6, 7, 18]. We considered, as other groups have stated, that specific targeted interventions to prevent these causes should be planned for in order to save the lives of these children [2, 8, 29]. Community mortality in vulnerable settings is common; however, currently there are no accurate estimates of the global burden of diseases at this level [8, 14, 30-32]. In this study, we determined that most home deaths in neonates occurred due to LRTI or heart diseases, while young infants (aged 1 to 12 months) are prone to die due to mild to moderate LRTI or pneumonitis due to milk aspiration. Comorbidities of older children play an important role, and severe nondetected bacterial pneumonia was frequent. Even though parental disease awareness seems to be low (low VA agreement in severe infections), before the fatal event, 80% of families had visited a health facility where the main health request was not resolved [7, 33].

Using this multidisciplinary approach, we were able to determine that vital registrations overestimated a SIDS diagnosis. In fact, of the 81 subjects with MITS performed, no immediate CoD could be found in only 3.

This study presents several limitations. First, findings may vary in scope and magnitude in other countries [8, 14]. Indeed, it would be key to elucidate the burden of community mortality through well-designed studies based on MITS or complete diagnosis autopsy in different regions. Second, bacterial pathogens were detected using only qPCR assays (no specific immunohistochemistry was performed), which limits our understanding of bacterial findings in the chain of events leading to death. Specific molecular approaches, such as immunohistochemistry alfa-lactalbumin measurement in lung tissue, could be helpful in determining the accurate burden of aspiration as an immediate CoD. Third, VA data recording may have parental recall bias, which was minimized by visiting families between 1 and 3 months after a child’s death [34, 35].

Implementing community-based studies with specimen collection in the developing world offers a novel approach for better assessment of accurate disease burden [8, 14, 19, 25]. The granular information provided in this study may help public health programs plan preemptive actions, allocate targeted resources, and engage the community to monitor and support those subgroups or families with young children at highest risk for death.

## Notes

***Acknowledgments.*** We thank the Ministerio Público de la Provincia de Buenos Aires, in particular, General Attorney Dr Julio Conte-Grand, Dr Francisco Pont Verges, and Dr Alejandra Attarian Mena. We also thank the Lomas de Zamora Judicial Morgue staff and its directors Dr Hernan Junod and Lucas Basanta. We express our gratitude to all of the beavered families who selflessly trusted in this study.

***Supplement sponsorship.*** This supplement is sponsored by MITS Surveillance Alliance Secretariat, led by RTI International, with funding from the Bill and Melinda Gates Foundation.

***Financial support.*** This publication is based on research funded by the Bill & Melinda Gates Foundation to F. P. P. (OPP1163671 and INV-009077).

***Potential conflicts of interest.*** F. P. P. reports grants and personal fees from Janssen (advisory board agreement) and Novavax, Inc (consulting agreement); personal fees from Bavarian Nordic A/S (consultancy agreement), Pfizer (advisory board agreement), Sanofi (advisory board agreement), Regeneron (consultant agreement), and Merck (consulting agreement); and grants from the Bill & Melinda Gates Foundation outside the submitted work. M. T. C. reports a grant award from the MITS Surveillance Alliance Secretariat. All remaining authors: No reported conflicts of interest. All authors have submitted the ICMJE Form for Disclosure of Potential Conflicts of Interest. Conflicts that the editors consider relevant to the content of the manuscript have been disclosed.
